# Prediction of the Physical Activity Level of Community-Dwelling Older Japanese Adults with a Triaxial Accelerometer Containing a Classification Algorithm for Ambulatory and Non-Ambulatory Activities

**DOI:** 10.3390/s23104960

**Published:** 2023-05-22

**Authors:** Shigeho Tanaka, Kazuko Ishikawa-Takata, Satoshi Nakae, Satoshi Sasaki

**Affiliations:** 1 Faculty of Nutrition, Kagawa Nutrition University, Saitama 350-0288, Japan; 2 Institute of Nutrition Sciences, Kagawa Nutrition University, Saitama 350-0288, Japan; 3 Department of Nutrition and Metabolism, National Institute of Health and Nutrition, National Institutes of Biomedical Innovation, Health and Nutrition, Osaka 566-0002, Japan; kt207460@nodai.ac.jp; 4 Department of Nutritional Science, Faculty of Applied Biosciences, Tokyo University of Agriculture, Tokyo 156-8502, Japan; 5 Human Augmentation Research Center, National Institute of Advanced Industrial Science and Technology, Chiba 277-0882, Japan; s.nakae@aist.go.jp; 6 Department of Social and Preventive Epidemiology, Graduate School of Medicine, The University of Tokyo, Tokyo 113-0033, Japan; stssasak@m.u-tokyo.ac.jp

**Keywords:** accelerometer, physical activity level, community-dwelling older adults, doubly labeled water, total energy expenditure

## Abstract

Accurate methods for the prediction of the total energy expenditure and physical activity level (PAL) in community-dwelling older adults have not been established. Therefore, we examined the validity of estimating the PAL using an activity monitor (Active style Pro HJA-350IT, [ASP]) and proposed correction formulae for such populations in Japan. Data for 69 Japanese community-dwelling adults aged 65 to 85 years were used. The total energy expenditure in free-living conditions was measured with the doubly labeled water method and the measured basal metabolic rate. The PAL was also estimated from metabolic equivalent (MET) values obtained with the activity monitor. Adjusted MET values were also calculated with the regression equation of Nagayoshi et al. (2019). The observed PAL was underestimated, but significantly correlated, with the PAL from the ASP. When adjusted using the Nagayoshi et al. regression equation, the PAL was overestimated. Therefore, we developed regression equations to estimate the actual PAL (Y) from the PAL obtained with the ASP for young adults (X) as follows: women: Y = 0.949 × X + 0.205, mean ± standard deviation of the prediction error = 0.00 ± 0.20; men: Y = 0.899 × X + 0.371, mean ± standard deviation of the prediction error = 0.00 ± 0.17.

## 1. Introduction

Adequate nutrition is essential to maintain health, particularly in older adults [1,2]. The energy requirement is an important factor, and accurate prediction of total energy expenditure (TEE) is necessary to establish the energy requirement. In most cases, TEE is predicted as the basal metabolic rate multiplied by the physical activity level (PAL) [3]. There are different types of methods to predict TEE, such as activity records, questionnaires, and accelerometry. Accelerometry appears to be the most useful, based on its accuracy and convenience [3]. However, its accuracy depends on the accelerometer and algorithm used [4,5,6,7,8]. Furthermore, accurate TEE and PAL prediction methods have not been established for older adults. Some research has indicated that accelerometers such as ActiGraph, with algorithms for young adults, tend to underestimate the intensity of various physical activities or TEE [9,10,11].

In Japan, the Active style Pro (ASP) is the most frequently used monitor to evaluate physical activity or TEE. The ASP has a classification algorithm for ambulatory and non-ambulatory activities [12] and predictive equations for the physical activity intensity of both types of activities [13]. The ASP is one of the most accurate tools for predicting the TEE of healthy Japanese adults. However, the ASP also tends to underestimate physical activity intensity in community-dwelling older adults [14,15] and TEE in frail older adults [16], elderly patients with type 2 diabetes mellitus [17] or those with COPD [18], and the prediction of TEE or PAL for community-dwelling older adults has not been validated. Nagayoshi et al. [15] published the results of multiple regression equations to predict the actual metabolic equivalent (MET) values from those obtained for young adults by the ASP.

In the present study, the PAL predicted by the ASP was validated with the doubly labeled water (DLW) method and the basal metabolic rate under free-living conditions in Japanese community-dwelling older adults. Furthermore, correction formulae for the predicted PAL were also developed for accurate prediction of the PAL.

## 2. Materials and Methods

### 2.1. Participants

Participants were recruited from the population of independent community-dwelling adults aged 65 to 85 years who joined the Itabashi Cohort Study in 2006 and 2011 and were selected so that the average number of step counts obtained with the Yamasa SW-200 matched that of the National Health and Nutrition Survey in each gender and age category. As a result, took a series of measurements of 81 older adults, and complete data were obtained from 69 participants (22 men and 47 women). The details of the sampling method are described elsewhere [19].

This study was conducted in accordance with the Declaration of Helsinki, and approved by the Ethics Committee of the National Institute of Health and Nutrition (20090327-02, approved on 27 March 2009) and National Institutes of Biomedical Innovation, Health and Nutrition (NIBIOHN 109-01, approved on 22 April 2020). The purpose and procedures of this study were explained in detail to the participants, and all gave their written informed consent.

### 2.2. Procedures

All measurements were conducted between February 2013 and May 2014. The participants visited the Tokyo Metropolitan Institute of Gerontology (TMIG), provided a baseline urine sample and received a dose of doubly labeled water (DLW) after anthropometric and basal metabolic rate (BMR) measurements. For the next 2 weeks, they were asked to maintain their normal lifestyle, to collect urine samples for TEE measurements, and to wear an accelerometer. They were asked to visit the TMIG again 2 weeks later, and their urine samples and accelerometers were collected. In addition, body mass was also measured.

### 2.3. Measurement of Energy Expenditure

The TEE under free-living conditions was measured using the DLW method [20]. The details are described elsewhere [19]. Briefly, participants were asked to provide a baseline urine sample on the first day before receiving a dose of water labeled with ^18^O (Taiyo Nippon Sanso Inc., Tokyo, Japan) and ^2^H (ISOTEC Sigma-Aldrich^®^ Merck MO, Burlington, MA, USA). Participants were asked to collect 8 urine samples during the next 2 weeks. The isotopic ratios were analyzed using isotope ratio mass spectrometry (DELTA Plus; Thermo Fisher Scientific, Waltham, MA, USA). The TEE was calculated using the A6 equation of Schoeller [21] and Weir’s equation [22]. The mean food quotient was calculated using Black’s equation [23] with the results from a dietary questionnaire and was used for the respiratory quotient.

Participants were asked to visit the TMIG after at least 12 h of overnight fasting on the morning of the first measurement day. After resting in the supine position for 30 min, at least 2 samples of expired gas were collected for 10 min each with Douglas bags. The O_2_ and CO_2_ concentrations were measured with a gas analyzer (AR-1, ARCO SYSTEM, Chiba, Japan). The gas volume was measured with a dry gas volume meter (DC-5, Shinagawa Corporation, Tokyo, Japan). The energy expenditure was calculated using Weir’s equation (13), and the mean value was taken as the BMR. The PAL was calculated as the TEE divided by the measured BMR.

### 2.4. Triaxial Accelerometer

A triaxial accelerometer, the Active style Pro HJA-350IT (ASP) (Omron Healthcare, Kyoto, Japan), was used to predict the MET values and measure step counts during the measurement period. The ASP can accurately predict the intensity of various types of physical activity [12,13] and the TEE [7,8] in adults under 60 years of age. Participants wore the ASP on the waist at the midline of the left thigh for 2 weeks during this study. They were asked to wear the accelerometer all day, except when bathing, swimming, or sleeping.

ASP HJA-350IT (74 mm × 46 mm × 34 mm and 60 g including battery) is equipped with 4 GB of memory consisting of Micro Electro Mechanical Systems-based accelerometers (LIS3LV02DQ; ST-Microelectronics, Geneva, Switzerland). Using ASP, 3-dimensional accelerations are obtained with a resolution of 3 mG and at a sampling rate of 32 Hz. Each of the three signals from the triaxial accelerometer was passed through a high-pass filter with a cutoff frequency of 0.7 Hz in order to remove the gravitational acceleration component from the signal [12]. The average of the absolute value of the accelerometer output of each of the three axes using acceleration signals is calculated over a 10 s interval. The data obtained by the ASP were imported into a PC via BiLink (Omron Healthcare, Kyoto, Japan).

MET values are calculated according to the ratio of synthesized unfiltered and filtered accelerations (ACunfil and ACfil) and the following 3 equations:

If ACfil < 29.9 mG,

Sedentary activity: MET = 0.8823 + 0.0351 × ACfil

If ACfil > 29.9 mG,

Then, if ACunfil/ACfil ≥ 1.16

Non-locomotive activity: MET = 1.3435 + 0.0196 × ACfil

Else if ACunfil/ACfil < 1.16

Locomotive activity: MET = 1.1128 + 0.0086 × ACfil

Further data processing was performed using the Excel macro program developed and distributed by the Japan Physical Activity Research Platform [24], in order to take into account the wear time, etc. Recorded data of ≥ 60 min of consecutive zero counts were defined as non-wear periods. Only days with a wear time ≥ 600 min were regarded as wear days and data of participants with at least 3 valid days were included in the analysis. Non-wear time was assigned a value of 0.9 METs based on the data of the calibration study [13]. The intensity of physical activity was divided into sedentary (≤0.5 METs), light (1.6–2.9 METs), moderate to vigorous (METs ≥ 3.0). Activity energy expenditure was estimated from minute-by-minute METs multiplied by the estimated resting metabolic rate (calculated as predicted BMR × 1.1). The BMR was predicted using the following equation for Japanese adults [25].

Predicted BMR = ((0.1238 + (0.0481 × body weight (kg)) + (0.0234 × stature (cm)) − (0.0138 × age) − gender *)) × 1000/4.186

* Men: 0.5473 × 1, women: 0.5473 × 2

The above predictive equation was derived from data of BMR measured at the National Institute of Health and Nutrition for Japanese men and women (71 men and 66 women). The PAL was also calculated from the MET values obtained from the ASP with diet-induced thermogenesis defined as 1/10 of the TEE [26] as follows:

Predicted TEE = (predicted BMR + predicted activity energy expenditure) × 10/9

Predicted PAL = predicted TEE/predicted BMR

MET values were adjusted using the regression equation in the elderly presented as Model 1 (adjusted METs = 1.191 × METs indicated by the ASP + 0.106) in Table 4 of Nagayoshi et al. [15].

### 2.5. Statistics

Data are presented as the mean and standard deviation. The differences between measured and predicted variables or between genders were compared using a *t* test. Pearson’s correlation and simple regression models were used to determine the relationships between measured and predicted variables. Significance levels for all tests were two tailed, at a level of 0.05. Statistical analyses were performed using Microsoft Excel SPSS for Windows V.27.0J (IBM Japan, Tokyo, Japan).

## 3. Results

The physical characteristics of the participants are summarized in Table 1. Values for step counts obtained with the ASP were higher than those with the Yamasa SW-200, which was used to select the participants of the present study.

**Figure 1:**
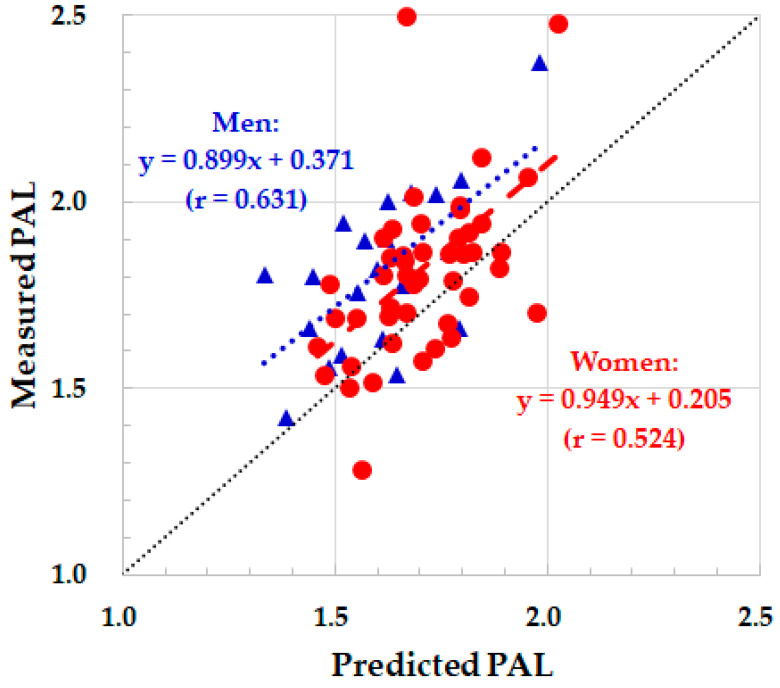
Relationship between measured and predicted PAL by the ASP without adjustment. Red closed circle and dotted regression line: women. Blue closed triangle and dotted regression line: men. Black dotted line: identity line.

The average PAL obtained with the DLW method and measured BMR was 1.82 ± 0.21 (Table 2). There was no significant gender difference in the PAL. The BMR was slightly overestimated in men and underestimated in women by the predictive equation. In contrast, the PAL was substantially underestimated by the ASP without adjustment, especially in women (Table 2 and Figure 1), although the predicted PAL was moderately correlated with the measured PAL (men: r = 0.631, women: r = 0.524). As a result, the TEE was significantly underestimated by the accelerometer in both genders, mainly due to the underestimation of the PAL. The correlation coefficients between the percentage of sedentary (0.164 in women and −0.154 in men), light (−0.153 in women and 0.116 in men), or moderate-to-vigorous physical activity (−0.099 in women and 0.184 in men) and the PAL were not significant.

**Figure 2:**
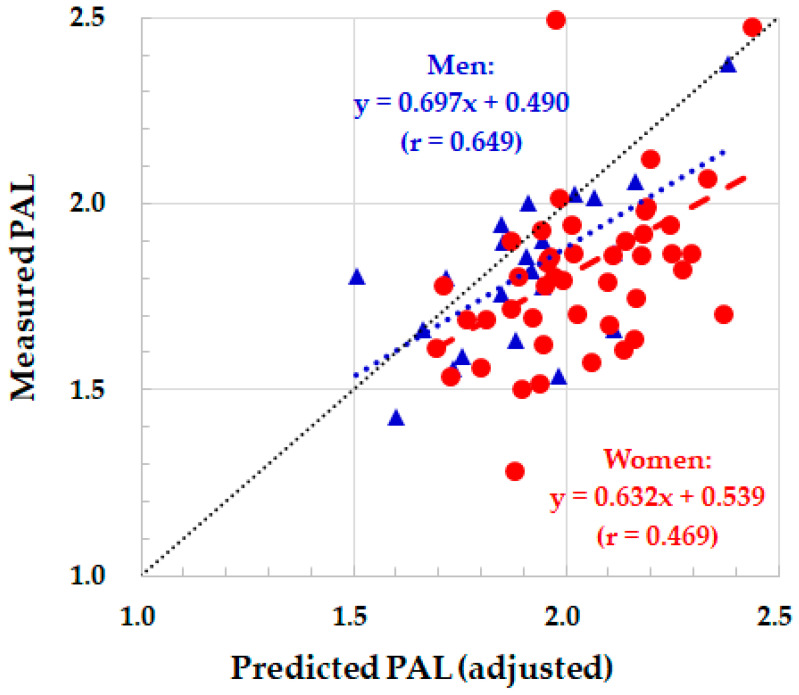
Relationship between measured and predicted PAL by the ASP adjusted with the regression equation of Nagayoshi et al. [15]. Red closed circle and dotted regression line: women. Blue closed triangle and dotted regression line: men. Black dotted line: identity line.

When the MET values adjusted with the regression equation of Nagayoshi et al. [15] were used, the PAL was overestimated, particularly in women (Table 2 and Figure 2). As a result, the PAL and TEE were significantly overestimated in both genders.

Therefore, the correction of the PAL obtained with the ASP was made using the regression equation between measured and predicted PAL (Figure 1). The equations for the prediction of the actual PAL (Y) from the PAL obtained with the ASP for young adults (X) are as follows:

Women: Y = 0.949 × X + 0.205, mean ± standard deviation = 0.00 ± 0.20

Men: Y = 0.899 × X + 0.371, mean ± standard deviation = 0.00 ± 0.17

The prediction error with the corrected PAL with the above equations and the predicted BMR was −107 ± 214 kcal/day for women and 102 ± 210 kcal/day for men. When the measured BMR was used instead of the predicted BMR, the prediction error was 3 ± 209 kcal/day for women and 6 ± 216 kcal/day for men.

## 4. Discussion

The results of the present study indicate that the ASP, which can accurately predict the TEE in adults under 60 years of age, underestimated the PAL and TEE in community-dwelling older adults. This is consistent with our previous results on the prediction of minute-by-minute physical activity intensity for community-dwelling older adults [14,15]. Therefore, adjustment of the MET values for older adults with a regression equation proposed by Nagayoshi et al. [15] was tried, but the adjusted MET values led to overestimation of the PAL.

Accurate prediction methods for older adults, including community-dwelling older adults who are independent in their daily life, have not been established. In addition to the lack of accurate predictive equations for the BMR, even the average PAL for such individuals has not been known. Among various methods to predict the TEE and/or PAL, accelerometry may be the most accurate and convenient. The ASP was proved to be the most accurate among activity monitors widely used for research or by consumers [7,8]. However, even the ASP tends to underestimate physical activity intensity, especially higher-intensity physical activity, in community-dwelling older adults. In the present study, the ASP containing an algorithm for young adults underestimated the PAL and TEE. The degree of underestimation was higher in men than in women. The degree of the underestimation is slightly smaller than that in Yamada et al. [27]. They used a different triaxial activity monitor (Actimarker, Panasonic, Tokyo, Japan) for relatively active older adults. According to the results by Murakami et al. [7,8], Actimarker provided a lower estimate of TEE and physical activity energy expenditure than the DLW method, while AS provided comparable results to the DLW method. Thus, the relative magnitude of the prediction error of the ASP and Actimarker is roughly consistent for younger and older adults.

Due to the underestimation of the MET values for older adults, we employed a regression equation to predict the actual MET values from those obtained with the ASP containing an algorithm for young adults (aged less than 60 years). However, the adjusted MET values led to overestimation of the PAL and TEE. The intercept of the equation was 0.106, so in the case that MET is 1.0, the adjusted MET value is 1.297. As a result, even for activity without movement, the predicted METs were much larger than 1.0 MET. Sedentary behavior (METs ≤ 1.5) occupied approximately two-thirds of all wear time. Thus, error in the adjustment using the regression equation for sedentary behavior is likely to be the main reason for the overestimation, although the correlation between the percentages of each category of physical activity intensity and PAL was not significant in men or women.

The method without or with adjustment of MET values for older adults led to underestimation or overestimation of the PAL and TEE. However, the correlations between the PAL obtained with the ASP without the above adjustment and the measured PAL were relatively good (men: r = 0.631, women: r = 0.524). Therefore, the correction of the PAL obtained with the ASP was made by using the regression equation. The prediction error of TEE was 3 ± 209 kcal/day for women and 6 ± 216 kcal/day for men when the measured BMR and the corrected PAL with the regression equation in the present study was used to predict the TEE. These standard deviations of the prediction error of the PAL and TEE were approximately 0.15 and 200 kcal/day, respectively. These prediction errors were much better than those reported previously [4,5,6,7,8]. Therefore, such a correction may be the best alternative to predict the PAL and TEE at present. The accuracy of the prediction of at least the PAL, and maybe the TEE as well, seems comparable to those for younger adults.

However, this correction does not contribute to accurate assessment of minute-by-minute METs. In many cases, the minute-by-minute MET value has been more frequently used than the PAL or TEE. Furthermore, while this correction is simple, it may not be applicable to community-dwelling older adults with various individual characteristics. Another problem is the lack of an accurate prediction equation for the BMR. In the algorithm of the ASP, the BMR was predicted by a predictive equation by Ganpule et al. [25], which can predict the BMR most accurately among available BMR equations for healthy Japanese individuals [28]. However, in addition to a slightly higher prediction error for healthy older adults, there are older adults with some diseases or disorders for whom the prediction errors tend to be large [16,17,18]. Thus, there are several factors that may contribute to the prediction errors for the TEE, which did not largely influence the findings of the present study. Our study has several limitations. Among them, the most important is that the results obtained may not be applicable to different populations, including those that vary in health status, age, and ethnicity.

The most important finding of the current study is that the ASP underestimates the PAL and TEE for community-dwelling older adults. However, there were correlations between the predicted and measured PAL and TEE. Therefore, we can capture the relative position of the PAL and TEE to some degree. Furthermore, the proposed correction equations may be useful for the accurate prediction of the PAL and TEE. However, the prediction errors presented in the present study are based on the regression equation obtained from the participants of this study, and if applied to a different sample, the prediction error should be larger. Therefore, validation studies of the equations for community-dwelling older adults are necessary. It seems that the PAL and TEE in older adults similar to those in the present study could be estimated with the same accuracy as in younger adults.

## 5. Conclusions

The accelerometer, which can accurately predict the TEE in adults under 60 years of age, underestimates the TEE and PAL in community-dwelling older adults. This is consistent with our previous results [14,15], which indicate that the accelerometer underestimates physical activity intensity in elderly individuals. On the other hand, adjustment with the regression equation indicated in Nagayoshi et al. [15] overestimated the PAL and TEE. Therefore, we provide a correction equation based on the obtained MET values for young adults, which can predict the PAL and TEE relatively accurately, with a standard error of approximately 0.15 and 200 kcal/day, respectively. This could be one of the best methods to predict PAL and TEE for community-dwelling older adults, although a validation study for such a population is needed.

## Figures and Tables

**Table 1:** Physical characteristics of participants.

Variable	Women	Men
Age, y	74 (6)	75 (5)
Stature, cm	149.4 (6.6)	162.0 (4.8)
Body weight, kg	52.5 (9.8)	62.7 (9.4)
Body mass index, kg/m^2^	23.5 (4.2)	23.9 (3.5)
Total steps, counts/day	5616 (2572)	5540 (2547)

**Table 2:** Measured and predicted energy expenditure and physical activity level.

Variable	Women	Men
Measured		
Basal metabolic rate, kcal/day	1021 (111)	1225 (157)
Total energy expenditure, kcal/day	1863 (316)	2219 (325)
Physical activity level	1.83 (0.24)	1.82 (0.23)
Predicted by the ASP		
Basal metabolic rate, kcal/day *	962 (137)	1279 (125)
Total energy expenditure, kcal/day *	1661 (269)	2049 (248)
Prediction error, kcal/day	−202 (246)	−170 (207)
Physical activity level	1.71 (0.13)	1.61 (0.15)
Prediction error	−0.12 (0.20)	−0.21 (0.17)
Predicted with Nagayoshi’s equation		
Total energy expenditure, kcal/day *	1957 (319)	2431 (312)
Prediction error, kcal/day	94 (221)	212 (217)
Physical activity level	2.04 (0.18)	1.90 (0.20)
Prediction error, kcal/day	0.21 (0.22)	0.09 (0.17)
Predicted with the regression equation		
Total energy expenditure, kcal/day *	1756 (277)	2321 (257)
Prediction error, kcal/day *	−107 (214)	102 (210)
Physical activity level	1.83 (0.13)	1.82 (0.14)
Prediction error	0.00 (0.20)	0.00 (0.17)

* Predicted using a predictive equation for BMR by Ganpule et al. [25].

## Data Availability

Data is available upon reasonable request and subject to ethical review.
